# Health implications resulting from the timing of elective cesarean delivery

**DOI:** 10.1186/1477-7827-8-68

**Published:** 2010-06-21

**Authors:** Raed Salim, Eliezer Shalev

**Affiliations:** 1 Department of Obstetrics and Gynecology, HaEmek Medical Center, Afula, Israel and Rappaport Faculty of Medicine, Technion, Haifa, Israel

## Abstract

**Background:**

The literature is nearly unanimous in recommending elective cesarean delivery at 39 weeks of gestation because of the lower rates of neonatal respiratory complications compared to 38 weeks. However, elective cesarean delivery at 39 weeks or more may have maternal and other fetal consequences compared to delivery at 38 weeks, which are not always addressed in these studies.

**Discussion:**

Between 38 and 39 weeks of gestation, approximately 10% - 14% of women go into spontaneous labor; meaning that a considerable number of women scheduled for an elective cesarean delivery at 39 weeks will deliver earlier in an unscheduled, frequently emergency, cesarean delivery. The incidence of maternal morbidity and mortality is higher among women undergoing non-elective cesarean deliveries than among those undergoing elective ones. Complications may be greater among women after numerous repeat cesarean deliveries and among older women. Other than reducing the frequency of non-elective cesarean deliveries, bringing forward the timing of elective cesarean delivery to 38 weeks, may occasionally prevent intrauterine fetal demise which has been shown to increase with increasing gestational age and to avoid other fetal consequences related to the emergency delivery. All these considerations need to be weighed against the medical and the economic impact of the increase in neonatal morbidity resulting from births at 38 weeks compared to 39 weeks.

**Summary:**

Until prospective randomized trials are conducted, we are unlikely to be able to precisely answer all risk:benefit questions as to the best timing of scheduled elective cesarean delivery. Older women, and women with numerous prior cesarean deliveries, are of particular concern. It is reasonable to inform the pregnant women of the risk of each of the above options and to respect her autonomy and decision-making.

## Background

The literature is nearly unanimous in recommending elective cesarean delivery at 39 weeks of gestation because of lower rates of neonatal respiratory complications compared to 38 weeks. However, elective cesarean delivery at 39 weeks or more may have maternal and other fetal consequences compared to delivery at 38 weeks, which are not always addressed in these studies. Delaying delivery for an additional week increases the time that the woman and her fetus is vulnerable to a number of unexpected complications and increases the proportion of women who will deliver by non-elective cesarean delivery rather than an elective one.

The outcome of this particular group of women is less addressed in the literature when discussing the advantages of elective cesarean deliveries since the majority of published studies on elective cesarean delivery exclude from statistical analysis women who delivered non-electively before the scheduled date of delivery. Other studies combined this cohort of patients with those that delivered electively so that it is impossible to isolate the contribution of non-elective delivery to the outcome. A design centered on the actual delivery route will allow investigators to distinguish between labored and unlabored cesarean deliveries. In studies limited to unlabored cesareans, women who present in labor before their scheduled date of delivery are, by definition, excluded. Excluding these women may overestimate potential benefits and also potential harms because the studies then cannot account for any effect that labor has on outcomes of interest. Landon et al [1] investigated maternal and perinatal outcomes among women who underwent an elective repeat cesarean delivery. Women who were designated for an elective cesarean delivery but presented in early labor were excluded from the study. The authors stated that exclusion from the study of women who presented in early labor and subsequently underwent repeated cesarean delivery probably lowered the risk of complications in the group of women undergoing elective repeated cesarean delivery [1].

Thomas et al reported that 10% of women go into spontaneous labor between 38 and 39 weeks of gestation [2]. Obstetrical data from 25,533 women delivered between the years 2003 to 2008 in our delivery ward (Obstetrics and Gynecology Department, HaEmek Medical Center in Afula, Israel, a university teaching hospital) revealed that 14% of ongoing pregnancies went into spontaneous labor between 38 to 39 weeks (unpublished data). The meaning of these numbers is that over 10% of elective cesarean deliveries scheduled to 39 weeks will likely convert to non-elective ones between 38 to 39 weeks.

A search of PubMed, MEDLINE, EMBASE, and Cochrane Library databases up to December 2009, did not detect any randomized controlled trial that compared the timing of elective cesarean delivery at 38 or 39 weeks and which investigated both perinatal and maternal outcomes.

In this report we address some of the maternal and perinatal consequences resulting from scheduling elective cesarean delivery at 39 weeks rather than 38 weeks. We claim that scheduling elective cesarean delivery at 38 weeks is a viable alternative with potential benefits particularly for older women and women with numerous prior cesarean deliveries.

## Discussion

Women assigned to an elective cesarean delivery may go into labor prior to the scheduled date of surgery. Laboring women might present during the early stages of labor, with or without membrane ruptures, or alternatively they may present during advanced stages of labor. Maternal and neonatal outcomes may be adversely affected when cesarean delivery is preceded by labor, even if labor is not advanced.

### Fetal and neonatal consequences

The implication of scheduling delivery to 39 weeks is that a proportion of elective cesarean deliveries will convert to non-elective ones, which may increase the risk of traumatic injury to the fetus/newborn [3]. The reported incidence of iatrogenic fetal trauma during cesarean delivery is 0.1% to 1.9% of births [4]. Several risk factors for fetal injury at the time of the cesarean delivery have been identified through various case reports. These include lack of surgical experience, labor with thinning of the lower uterine segment exposing the fetus to injury with the scalpel, and a lack of amniotic fluid secondary to rupture of the membranes making the underlying fetal parts more accessible [5,6]. Fetal lacerations, finger injuries and amputations, penetrating brain injuries, skull fractures and long bone fractures have all been reported from the use of the scalpel or scissors at the time of cesarean delivery [3]. Although traumatic delivery is still associated with cesarean delivery, it is almost unheard of with elective cesarean delivery of the vertex fetus at term [3].

In the term breech trial, 6% of women who were assigned to a planned cesarean delivery, delivered vaginally because cesarean delivery was not possible due to imminent vaginal delivery [7]. Delaying an elective cesarean delivery scheduled for breech presentation may expose some of the fetuses to preventable morbidity and mortality associated with vaginal breech delivery in cases where vaginal delivery is imminent at admission.

An accumulative increased risk of intrauterine fetal death has been reported with increasing gestational age. The timing of fetal death for stillborn infants born between 23 and 40 weeks is evenly distributed with nearly 5% of all stillbirths occurring per week of gestation [8]. This is important when considering all stillborn infants at 38 weeks and beyond, where significant complications of prematurity would be very rare if only these fetuses had simply been delivered earlier. Furthermore, it has been reported that a fairly stable rate of fetal death of 0.6 per 1000 live births occurs from 33 weeks to 39 weeks of gestation. However, at 39 weeks, the rate increases significantly to 1.9 per 1000 live births [9]. De la Vega and coworkers in a mixed risk population with unrestricted access to testing for fetal wellbeing and sonographic evaluations concluded that, despite intensive surveillance, they were still unable to reduce the rate of fetal death. The investigators suggested that this is probably due to occurrence of acute placental and cord accidents that cannot be detected through antenatal fetal surveillance and are simply unavoidable [10]. The sudden death of a fetus in utero has medical, social and economic implications. It is particularly tragic when it occurs shortly before the expected date of delivery.

### Maternal consequences

Hansen et al reported in a cohort study that a significant reduction in neonatal respiratory morbidity may occur if elective caesarean delivery is postponed to 39 weeks. Carrying out elective cesarean deliveries at a later gestational age resulted in higher rates of laboring cesarean deliveries since some women went into spontaneous labor. Twenty-five percent of women entered labor before the 39^th ^week in their cohort. They also stated that compared with elective cesarean deliveries, laboring cesarean deliveries may carry an increased risk of complications such as uterine rupture, infections, or even maternal mortality [11].

Severe maternal morbidities including deep vein thrombosis, pulmonary embolism, amniotic fluid embolism, puerperal infection, severe hemorrhage, uterine rupture or inversion and intestinal obstruction have been reported to be significantly more frequent in non-elective than in elective cesarean deliveries. Operative interventions after delivery were also significantly more frequent after a non-elective cesarean delivery [12]. Operator experience and the emergency nature of the cesarean delivery were found to be risk factors for bladder injury during cesarean delivery [13].

The overall risk of uterine rupture among women who go into spontaneous labor is higher compared to women who had an elective cesarean delivery. The risk of rupture is greater among parous women with multiple prior cesarean deliveries, a situation commonly encountered in some regions [14]. Other than maternal morbidity, a ruptured uterus carries a greater risk for perinatal morbidity and mortality [14].

Although uterine rupture occurs primarily among women during a trial of labor, women assigned to an elective cesarean delivery will occasionally present in the advanced stages of labor before their scheduled date of operation.

Failed intubation and pulmonary aspiration are the leading causes of anesthesia related maternal morbidity and mortality. Fasting for a period of six to eight hours is recommended before an elective cesarean delivery [15]. Women scheduled for an elective cesarean delivery and who go into spontaneous labor may present while not in the fasting state. Performing an immediate operation because the woman is in labor may increase anesthesia related morbidity and mortality. Alternatively, delaying the procedure six to eight hours may increase the likelihood of progression into advanced labor which may further complicate the operation. Furthermore, in women whose indication for a cesarean delivery is human immunodeficiency virus infection or genital herpes, the risk of neonatal infection may increase if abdominal delivery is delayed.

Women with severe urinary incontinence have a marked deterioration in their quality of life, most substantially curtail activities, many become homebound, and for some, urinary incontinence is the defining event that prompts nursing home admission. In the United States each year, an estimated 135,000 women undergo surgery for urinary incontinence [16]. An estimate of direct costs for urinary incontinence in the United States has been reported to be $16 billion per year [17]. Given the substantial public health burden of pelvic floor disorders, much research has been focused on identifying risk factors, especially modifiable risk factors, for the development of pelvic floor disorders. Retrospective and cross-sectional studies implicate childbirth as a major risk factor for urinary incontinence in younger women [18]. Whether, and to what degree, cesarean delivery may protect child-bearing women from developing urinary incontinence is an unresolved issue. Several prospective studies evaluated the risk of postpartum urinary incontinence by delivery type, grouping all cesarean deliveries together and reported inconsistent results. In one study, however, elective cesarean deliveries were separated form non-elective ones. Chin et al assessed the impact of delivery on the pelvic floor and to what degree cesarean deliveries could prevent pelvic floor injury. Five hundred thirty nine women were divided into three groups according to the delivery method adopted: elective cesarean delivery, non-elective cesarean delivery, and vaginal delivery. Only elective cesarean delivery was protective. They concluded that the key to the best protection against postpartum urinary incontinence seems to lie in the timing of the cesarean delivery; that is, the cesarean delivery has to be performed before labor or uterine contractions have commenced [19].

Delaying delivery until 39 weeks may increase the risk for pregnancy complications such as gestational hypertension, preeclampsia, and eclampsia that are known to increase in incidence from 37 to 43 weeks when calculated according to ongoing pregnancies [20].

Data suggests an increased risk of maternal mortality with non-elective cesarean deliveries as compared with elective ones. The Report on Confidential Enquiries into Maternal Deaths, 1997 to 1999, reported a significantly higher maternal mortality rate with emergency and urgent cesarean deliveries [21]. Another publication reporting on deliveries in Israel between 1984 and 1992 compared maternal mortality among vaginal deliveries, emergency and elective cesarean deliveries. The authors reported maternal mortality rates of 2.8, 3.6, and 30 per 100,000 deliveries for elective cesarean delivery, vaginal delivery, and emergency cesarean delivery, respectively [22].

### Advance maternal age

More women are postponing pregnancy into the fourth and fifth decades of life for a variety of reasons. Advanced maternal age, traditionally defined as over 35 years, has been associated with increased obstetric morbidity and interventions.

Older women are more likely to have elective cesarean deliveries [23]. The risk for severe complications in non-elective cesarean deliveries is even higher among older women than in younger ones [12]. Furthermore, perinatal complications are reported to be higher among this population [24]. Intrauterine fetal death and perinatal mortality are significantly higher in older women even after excluding deaths due to congenital malformations and adjusting for existing illnesses or pregnancy complications [25]. The highest rate of stillbirth was reported to occur among older women after 38 weeks of gestation [26].

### Health Care Provider Type and Professional Resources

The availability of resources, such as operating rooms and staff, may influence a health care provider's decision regarding when to schedule the date of the elective cesarean delivery. Non-elective cesarean deliveries, which by definition are poorly timed, may result in a patient that presents in the non fasting state, at a time that the hospital is staffed with less experienced surgeons and anesthetists whose skills are further compromised due to demanding working hours. All these factors present additional challenges to the patients' safety. One of the advantages of scheduled operations is the greater ease of balancing staffing levels with clinical volume. Inadequate levels of staffing, as well as fatigue among health care providers, may contribute to increased patient morbidity [27,28].

## Summary

Multiple chance events may influence outcome. For example, an elective cesarean delivery at 38 weeks may result in the delivery of an iatrogenically premature infant at risk for respiratory morbidity. On the other hand, delaying delivery to 39 weeks may result in an unexplained stillbirth, or spontaneous onset of labor with intrapartum complications that may compromise maternal and neonatal well-being. Decision analysis is a quantitative methodology for evaluating competing strategies under conditions of uncertainty.

According to our data, 14% of all women booked for an elective cesarean delivery at exactly 39 weeks and 0 days, would be expected to go into spontaneous labor between 38 to 39 weeks (unpublished data). For an average hospital with 4500 births a year, such as ours, and a 10% elective cesarean delivery rate, scheduling delivery at 38 weeks rather than 39 weeks will result in an additional 10 neonates with respiratory morbidity a year, assuming an additional 2% neonatal morbidity for those delivered at 38 weeks [29]. On the other hand, 63 non-elective cesarean deliveries will be prevented. In fact, since it is not feasible to book all women to exactly 39 weeks and 0 days, particularly in public medical centers, the number of non-elective operations that would be prevented may actually be higher. Other than decreasing the risk of non-elective cesareans, scheduling elective cesarean deliveries to 38 weeks may prevent cases of fetal death especially among older women.

Until prospective randomized trials are conducted, we are unlikely to be able to precisely answer all risk:benefit questions as to the best timing of scheduled elective cesarean delivery. We believe that if dating is confirmed with an ultrasound study prior to 20 weeks of gestation, scheduling cesarean delivery to 38^+0-6 ^weeks may be another reasonable and alternative option to 39 weeks. This is particularly true among a selected group of women, namely older women and women where a complicated cesarean delivery is anticipated. It is reasonable to inform women of the risks entailed with each of the above options. The clinician's role should be to provide the best evidence-based counseling possible to the woman, and to respect her autonomy and decision-making.

## Competing interests

The authors declare that they have no competing interests.

## Authors' contributions

RS and ES contributed to the formation, drafting and editing of this article. Both authors read and approved the final manuscript.

## Authors' information

RS is a senior obstetrician, head of delivery ward, Department of Obstetrics and Gynecology HaEmek Medical Center, Afula, Israel; ES is a professor and associate Dean, Rappaport Faculty of Medicine Technion, Israel Institute of Technology, Chairman, Department of Obstetrics and Gynecology HaEmek Medical Center, Afula, Israel.
